# Burden of chronic kidney disease in resource-limited settings from Peru: a population-based study

**DOI:** 10.1186/s12882-015-0104-7

**Published:** 2015-07-24

**Authors:** Elizabeth R. Francis, Chin-Chi Kuo, Antonio Bernabe-Ortiz, Lisa Nessel, Robert H. Gilman, William Checkley, J. Jaime Miranda, Harold I. Feldman, CRONICAS Cohort Study Group

**Affiliations:** Penn State College of Medicine, Hershey, PA USA; Office of Global Health Education, Weill Cornell Medical College, New York, NY USA; Johns Hopkins Bloomberg School of Public Health, Baltimore, MD USA; CRONICAS Center of Excellence in Chronic Diseases, Universidad Peruana Cayetano Heredia, Av. Armendáriz 497, Miraflores, Lima 18, Peru; Kidney Institute and Division of Nephrology, Department of Internal Medicine, China Medical University Hospital and College of Medicine, China Medical University, Taichung, Taiwan; School of Public Health and Administration, Universidad Peruana Cayetano Heredia, Lima, Peru; Center for Clinical Epidemiology and Biostatistics, Perelman School of Medicine, University of Pennsylvania, Philadelphia, PA USA; Department of International Health, Bloomberg School of Public Health, Johns Hopkins University, Baltimore, USA; Área de Investigación y Desarrollo, Asociación Beneficia PRISMA, Lima, Peru; Division of Pulmonary and Critical Care, School of Medicine, Johns Hopkins University, Baltimore, MD USA; Department of Medicine, School of Medicine, Universidad Peruana Cayetano Heredia, Lima, Peru; Departments of Biostatistics and Epidemiology, Perelman School of Medicine, University of Pennsylvania, Philadelphia, USA; Department of Medicine, Perelman School of Medicine, University of Pennsylvania, Philadelphia, USA

**Keywords:** Chronic kidney disease, Prevalence, Chronic diseases

## Abstract

**Background:**

The silent progression of chronic kidney diseases (CKD) and its association with other chronic diseases, and high treatment costs make it a great public health concern worldwide. The population burden of CKD in Peru has yet to be fully described.

**Methods:**

We completed a cross sectional study of CKD prevalence among 404 participants (total study population median age 54.8 years, 50.2 % male) from two sites, highly-urbanized Lima and less urbanized Tumbes, who were enrolled in the population-based CRONICAS Cohort Study of cardiopulmonary health in Peru. Factors potentially associated with the presence of CKD were explored using Poisson regression, a statistical methodology used to determine prevalence ratios.

**Results:**

In total, 68 participants (16.8 %, 95 % CI 13.5–20.9 %) met criteria for CKD: 60 (14.9%) with proteinuria, four (1%) with eGFR <60mL/min/1.73m2 , and four (1%) with both. CKD prevalence was higher in Lima (20.7 %, 95 % CI 15.8–27.1) than Tumbes (12.9 %, 95 % CI 9.0–18.5). Among participants with CKD, the prevalence of diabetes and hypertension was 19.1 % and 42.7 %, respectively. After multivariable adjustment, CKD was associated with older age, female sex, greater wealth tertile (although all wealth strata were below the poverty line), residence in Lima, and presence of diabetes and hypertension.

**Conclusions:**

The high prevalence rates of CKD identified in Lima and Tumbes are similar to estimates from high-income settings. These findings highlight the need to identify occult CKD and implement strategies to prevent disease progression and secondary morbidity.

**Electronic supplementary material:**

The online version of this article (doi:10.1186/s12882-015-0104-7) contains supplementary material, which is available to authorized users.

## Background

Owing to the rising global epidemic of diabetes [1], hypertension [2], and obesity [3], chronic kidney disease (CKD) has become a worldwide public health problem with a substantial economic burden. For instance, the World Health Organization estimated a high cost-effectiveness ratio for dialysis, roughly $108,600 USD per disability-adjusted life-year [4, 5]. CKD further increases diabetes and hypertension-related complications, including cardiovascular risk and all-cause mortality [4, 6, 7]. In addition, due to major constraints on the availability of healthcare resources, CKD may impose a tougher challenge on low- and middle-income countries (LMIC). Understanding the epidemiology of CKD in LMIC is a fundamental step to addressing the burden of CKD and will guide disease surveillance, screening, prevention activities as well as healthcare resource allocation.

Given its diverse range of socio-economic trends and climatic and geographical zones, Peru provides a unique opportunity in which to assess CKD burden [8]. In 2013, approximately 65 % of the Peruvian population has some form of health insurance [9], but only an estimated 30 % of all Peruvians are able to access renal replacement therapy [10]. Another emerging challenge to current vulnerable health systems in Peru is its rapid population growth, which is reflected by changes in age structure and urbanization [11, 12]. However, as Peru is experiencing a marked demographic and epidemiologic transition with a rising prevalence of cardiovascular disease risk factors such as obesity, hypertension and type-2 diabetes, related illnesses such as CKD are also likely to be rising in prevalence [13, 14]. This scenario highlights the importance of detection of CKD and implementation of interventions that stem its progression toward end-stage disease.

In Peru, as in many other countries in Latin America, the information related to the epidemiology of CKD and end-stage renal disease is limited. Early hospital-derived data from 1990 suggested the prevalence of CKD in Lima to be only 12.2/100,000 people [15]. Since 1991, the Latin American Dialysis and Renal Transplant Registry has collected data from 20 countries and reported a high utilization of renal replacement therapy [16]. These data suggest that the burden of CKD has been under-appreciated and the need to design CKD surveillance programs as well as appropriate end-stage renal disease management [17, 18].

**Fig. 1:**
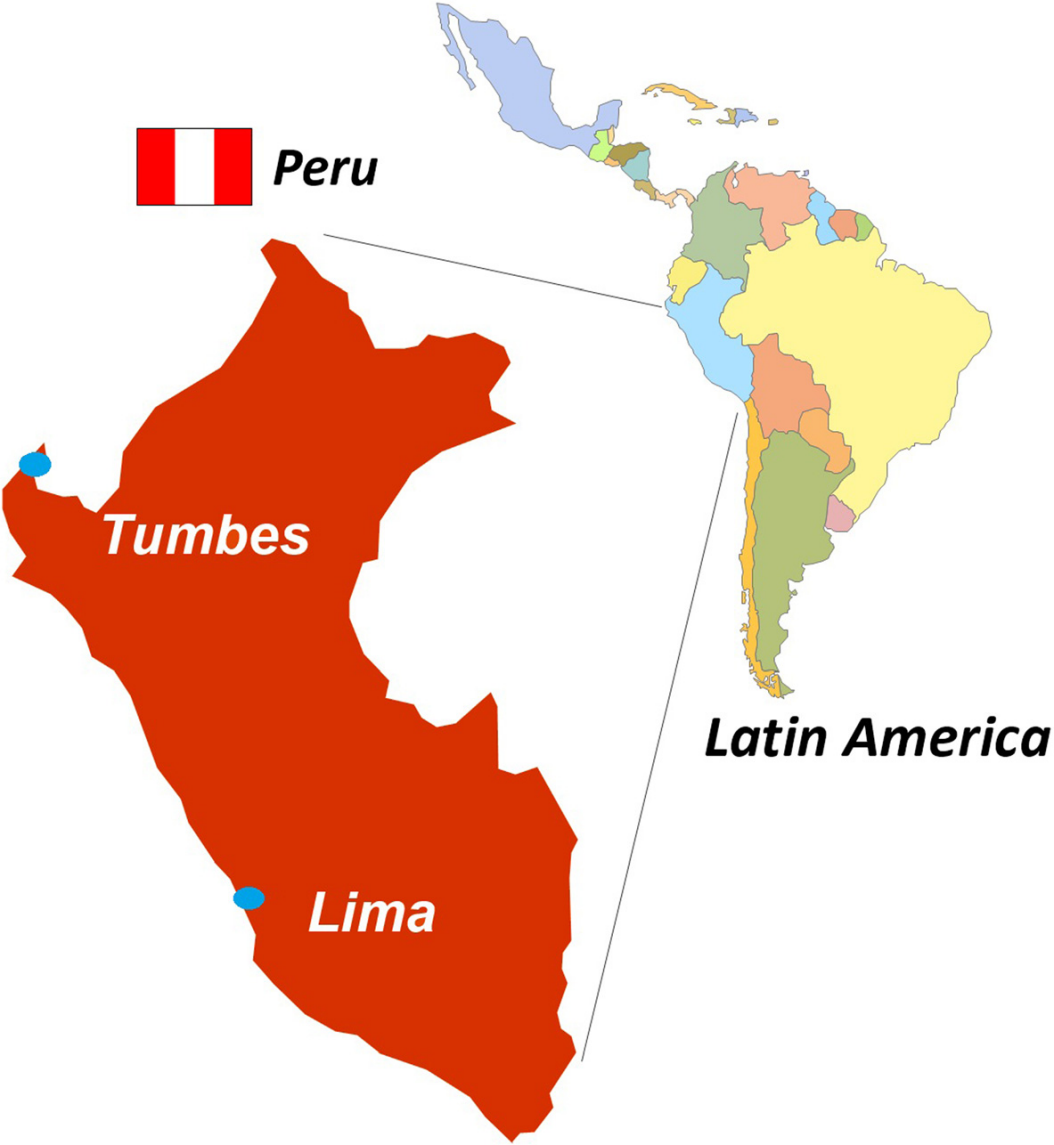
Map of study sites in Peru. Copyright permission. One of the authors (C-CK) created this image using ConceptDraw PRO software

The CRONICAS cohort study is a general population-based longitudinal study in Peru assessing cardiopulmonary risk factors [19]. This study examined CRONICAS study participants identified at two of the three study sites, Lima and Tumbes (Fig. 1), to estimate the prevalence of CKD and its potential risk factors.

## Methods

### Study design and setting

This cross-sectional study collected baseline CKD marker data in a random subsample of the ongoing CRONICAS cohort study [19]. Data were collected from February to May 2011 in the highly-urbanized site of Pampas de San Juan de Miraflores, Lima, Peru with 60,000 inhabitants in 4 km^2^, and the semi-urban site of Tumbes with 20,000 inhabitants in 80 km^2^. Lima, the country’s capital, is located in the central west coast region of Peru and Tumbes is located along the northwest coastline. Both regions have undergone different degrees of urbanization, including urban-industrial development [20].

### Participants, recruitment, and ethics

Using the most updated local census data, study participants were identified using a single-stage sampling method stratified by sex and age (35–44; 45–54; 55–64; ≥ 65 years old) [19]. Participants were included if they were full-time residents in the area, capable of understanding the study’s procedures, and able to provide informed consent. Patients were excluded if they were pregnant or unable to sit upright or lay down, precluding measurement acquisition. Participants were selected until each age and sex category was filled. Only one member per household was enrolled in the study.

Trained fieldworkers went door-to-door to contact selected participants. After discussing the parent study protocol and receiving informed consent, participants were scheduled to gather anthropometric measurements. At this appointment, a sub-sample of participants were approached and invited to participate in the CKD ancillary study and enrolled after obtaining verbal informed consent. The study’s protocol was reviewed and approved by the Institutional Review Boards of Universidad Peruana Cayetano Heredia and University of Pennsylvania.

### Data collection

The protocol for the CRONICAS Cohort Study has been published elsewhere [19]. Briefly, each participant completed a fieldworker-administered questionnaire to provide information on socio-demographic and lifestyle data including age, sex, education level, work status, alcohol consumption, smoking habits, previous medical history, and medication use. The questionnaire was based on the STEPS approach for surveillance of non-communicable diseases [21]. Trained fieldworkers measured height and weight from all participants during the clinic visit [19]. Each measurement was taken three times, and the average of the three was used for analyses. In addition, three systolic (SBP) and diastolic (DBP) blood pressure measurements were acquired using an automated monitor (OMRON HEM-780) [19]. The second and third blood pressure measurements were averaged for the analysis.

A trained laboratory technician obtained fasting venous blood and urine samples. Whole blood and plasma were collected in ethylenediaminetetraacetic acid (EDTA) tubes and sodium fluoride/EDTA tubes, respectively. Urine was collected in 15 mL containers. Blood serum and urine specimens were maintained at 4–8 °C for two weeks and then moved to a storage facility where the urine was aliquoted into 4 vials (1.5 mL each). All samples were then stored at −20 °C until laboratory analyses were performed. All samples were analyzed in a single laboratory. Assay quality was checked against regular external standards and internal duplicate assays and monitored by BioRad (http://www.biorad.com).

Details pertaining to the measurement of plasma glucose, serum insulin, hemoglobin A1C, total cholesterol and high-density lipoprotein (HDL)-cholesterol are outlined in the parent study protocol. [18] High-sensitivity C reactive protein was measured using Latex (Tina-quant CRP-HS Roche/Hitachi analyzer, Indianapolis, IN, USA). Serum and urine creatinine were measured by the modified kinetic Jaffé method. The enzymatic method was standardized to the IFCC’s isotope dilution mass spec (IDMS) method. Urine protein was determined via turbidimetry as measured by the Sequoia-Turner digital fluorometer.

### Study variables

The glomerular filtration rate, a measure of kidney function, was estimated using the CKD-Epidemiology Collaboration equation using serum creatinine as a filtration marker [22]. To ensure consistency across studies, CKD was defined as estimated glomerular filtration rate (eGFR) <60 ml/min/1.73 m^2^ or proteinuria (protein-creatinine ratio) ≥150 mg/g creatinine, or both, based on the latest Kidney Disease Improving Global Outcomes (KDIGO) guideline [4].

Diabetes mellitus was defined as fasting plasma glucose ≥126 mg/dL or self-reported physician diagnosis or use of anti-diabetic medications [23]. Hypertension was defined as a systolic blood pressure (SBP) of ≥140 mmHg, a diastolic blood pressure (DBP) of ≥90 mmHg, receipt of anti-hypertensive therapy at the time of enrollment, or self-report of a diagnosis by a physician [24].

Insulin resistance was assessed using the homeostasis model assessment (HOMA-IR) originally described by Mathew et al. [25] The Framingham risk score (FRS) was calculated from National Cholesterol Education Program (NCEP) Adult Treatment Panel (ATP) III algorithm and based on six cardiovascular risk factors: age, gender, total cholesterol, HDL-cholesterol, systolic BP and smoking status [26]. A 10-year risk of coronary events was divided into three levels of risk: low (<10 %), intermediate (10–20 %), and high (>20 %) [26].

Socioeconomic status was assessed using a wealth index based upon current occupation, household income, assets and household facilities [27]. Smoking was categorized as current, former, or never. Alcohol consumption was categorized into non-current and current drinkers. Body mass index (BMI) was calculated as weight in kilograms divided by height in meters squared.

### Statistical analysis and model selection

Categorical variables were described as proportions, and continuous variables were described as median with interquartile range (IQR). Differences between CKD and non-CKD participants were examined using Chi-square tests for categorical variables or Student t-tests for continuous variables. Both eGFR and protein-creatinine ratio were right skewed and, thus, log-transformed for linear regression. The 2-sided statistical significance level was set at α = 0.05.

Multivariate Poisson regression was performed to calculate prevalence ratios [28] and to assess the relationship between CKD and associated potential risk factors. These models were initially adjusted for sociodemographic and lifestyle variables including age, education, smoking habits, alcohol consumption and BMI, followed by adjustments for comorbidities including diabetes mellitus, hypertension, and C-reactive protein. Exploratory analyses were conducted within subgroups of the following covariates: age, sex, obesity, smoking, alcohol, total cholesterol, hypertension, diabetes mellitus, and Framingham risk score. All statistical analyses were conducted using STATA version 11.0 statistical software (StataCorp LP, College Station, TX, USA). It should be noted that exact logistic regression was also used to for univariate analysis and yielded results consistent with those found with Poisson regression.

## Results

### Population characteristics

The overall participation rate in the CRONICAS parent study was 62.9 %. A total of 404 adults, mean age 54.9 years [SD 13.0], 50.2 % male, were invited and agreed to participate in this sub-study of CKD. All 404 participants, 203 from Lima and 201 from Tumbes, completed a questionnaire, a clinical examination and laboratory tests. Overall, the prevalence of diabetes and hypertension were 9.9 % (40/404, 95 % CI 7.4–13.3 %) and 29.2 % (118/404, 95 % CI 25.1–34.0 %), respectively.

### Prevalence chronic kidney disease

The prevalence of CKD was 16.8 % (68/404, 95 % CI 13.5 %–20.9 %). Participants with CKD tended to be older, female (69.1 %), consumers of alcohol (98.5 %), less educated, and with more comorbid conditions, including diabetes and hypertension (Table 1).

**Table 1:** Characteristics of participants with and without chronic kidney disease (CKD) in CRONICAS-CKD pilot study

	No CKD	CKD	*p*-value
	*n* = 336, 83.1 %	*n* = 68, 16.8 %	
	Median (IQR) or	Median (IQR) or	
	n (%)	n (%)	
*Sociodemographics*			
Age, year	53.5 (43.6–63.2)	59.6 (50.5–69.2)	<0.01
Male, n (%)	182 (54.2)	21 (30.9)	<0.01
Location, n (%)			
Lima	161 (47.9)	42 (61.8)	0.04
Tumbes	175 (52.1)	26 (38.2)	
Education (yrs), n (%)			<0.01
Primary or less	138 (41.1)	41 (60.3)	
Secondary	139 (41.4)	14 (20.6)	
Higher than secondary	59 (17.6)	13 (19.1)	
Wealth index, n (%)			0.17
Low	67 (19.9)	7 (10.3)	
Medium	134 (39.9)	30 (44.1)	
High	135 (40.2)	31 (45.6)	
Smoking, n (%)			0.20
Never	158 (47.0)	40 (58.8)	
Former	132 (39.3)	20 (29.4)	
Current	46 (13.7)	8 (11.8)	
Alcohol, n (%)			0.56
Yes	150 (44.6)	33 (48.5)	
No	186 (55.4)	35 (51.5)	
*Comorbidities and laboratory findings*			
Diabetes Mellitus, n (%)	27 (8.0)	13 (19.1)	<0.01
Fasting glucose, mg/dL	93 (87–101)	96 (87–109)	<0.01
HbA1c, %	5.7 (5.5–6.0)	5.9 (5.6–6.4)	<0.01
Hypertension, n (%)	89 (26.5)	29 (42.7)	<0.01
Obesity, n (%)	112 (33.3)	22 (32.4)	0.88
Body mass index, kg/m^2^	28.1 (25.3–31.3)	27.3 (24.3–31.1)	0.55
Total cholesterol, mg/dL	201 (178.5–227)	203 (184–247.5)	0.06
Urine creatinine, mg/dL	100.8 (68.1–145.2)	55.4 (39.6–104.4)	<0.01
Protein-creatinine ratio, mg/g	79.9 (59.7–103.7)	204.6 (169.8–324.6)	<0.01
eGFR , ml/min/1.73 m2	99.8 (89.1–109.2)	98.1 (80.5–107.3)	<0.01
hs-CRP mg/L	2.2 (1.0–4.3)	2.0 (1.3–4.2)	0.65
HOMA-IR	1.96 (1.15–3.35)	2.18 (1.12–3.67)	0.01
HOMA-β	102.1 (59.8–162.8)	93.2 (55.7–136.1)	0.80
Framingham risk score, n (%)			0.15
Low	166 (49.4)	25 (36.8)	
Intermediate	100 (29.8)	24 (35.3)	
High	70 (20.8)	19 (27.9)	

*eGFR* estimated glomerular filtration rate, *HbA1c* hemoglobin A1c, *HDL* high-density lipoprotein, *HOMA-IR* homeostatic model assessment- insulin resistance, *HOMA-β* homeostatic model assessment- β cell function, *hs-CRP* high sensitivity C-reactive protein, *LDL* low-density lipoprotein

Among the 68 participants meeting the definition of CKD, 60 had isolated proteinuria, 4 had isolated impaired estimated glomerular filtration rate (eGFR <60 ml/min), and 4 had both proteinuria and impaired eGFR. Among participants with reduced eGFR (*n* = 8), mean eGFR was 40.1 ml/min (range 15.0 – 57.7 ml/min). Among participants with proteinuria (*n* = 64), the mean protein/creatinine ratio was 345.71 (range 151.5 – 1840.9 mg/g creatinine).

### Factors associated with CKD

In unadjusted Poisson analysis, older age, females, residents of Lima, and those with less education, greater levels of insulin resistance, diabetes, and hypertension were associated with higher probability of having CKD (Table 2).

**Table 2:** Factor associated with chronic kidney disease

	Crude prevalence ratio	Adjusted prevalence ratio
	(95% CI)	(95% CI)^a^
Age change by 10 years	1.47 (1.20–1.80)	1.48 (1.18–1.87)
Sex		
Female	1 (reference)	1 (reference)
Male	0.38 (0.22–0.66)	0.34 (0.19–0.62)
Education		
High school or less	1 (reference)	1 (reference)
More than high school	0.71 (0.41–1.24)	–
Wealth Index		
Low	1 (reference)	1 (reference)
Medium	2.14 (0.89–5.13)	2.57 (1.02–6.44)
High	2.20 (0.92–5.25)	2.64 (1.04–6.67)
Study site		
Lima	1 (reference)	1 (reference)
Tumbes	0.57 (0.33–0.97)	0.54 (0.30–0.96)
Diabetes		
No	1 (reference)	1 (reference)
Yes	2.71 (1.32–5.56)	2.21 (1.03–4.78)
Hypertension		
No		
Yes	2.06 (1.20–3.53)	1.38 (0.75–2.54)
HOMA-IR	1.09 (1.01–1.16)	–
hs-CRP change by 5 mg/L units	1.04 (0.86–1.27)	–
Framingham risk score classification		
Low	1 (reference)	–
Intermediate	1.59 (0.86–2.94)	–
High	1.80 (0.93–3.48)	–

^a^Models to calculate adjusted prevalence ratios (*n* = 404, no missing values) were adjusted, where appropriate, for all of these variables: age, sex, wealth index, study site, diabetes, and hypertension

In multivariable Poisson analysis, age, sex, higher wealth index, Lima residence, and diabetes were all independently associated with CKD (Table 2). Males had a 66 % lower prevalence of CKD than females and participants at the Tumbes site had a 46 % lower prevalence than among those recruited at the Lima site. Diabetes was associated with doubling prevalence of CKD. It is noted that HOMA, hs-CRP, and Framingham Risk Score were not significant in the final model.

### Exploratory stratified analysis by sex and geographical site

**Fig. 2:**
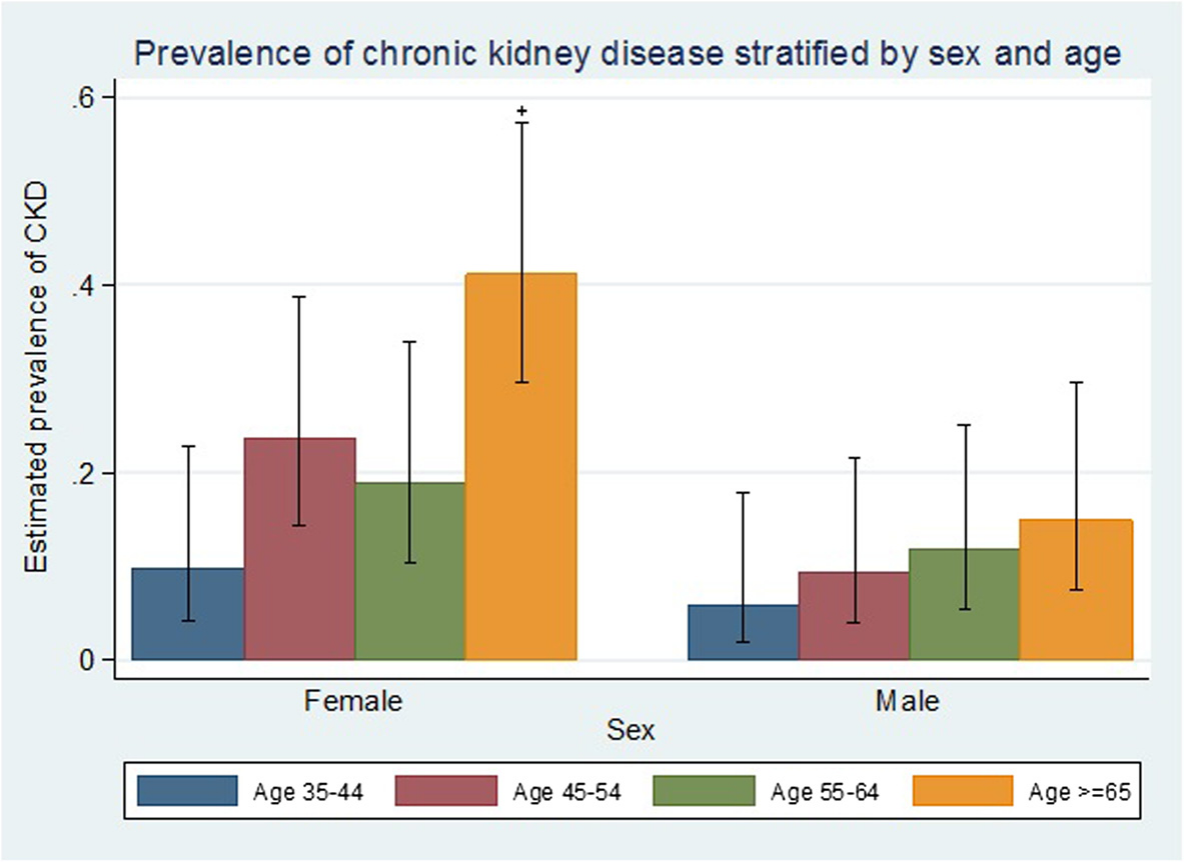
Prevalence of chronic kidney disease stratified by sex and age

**Fig. 3:**
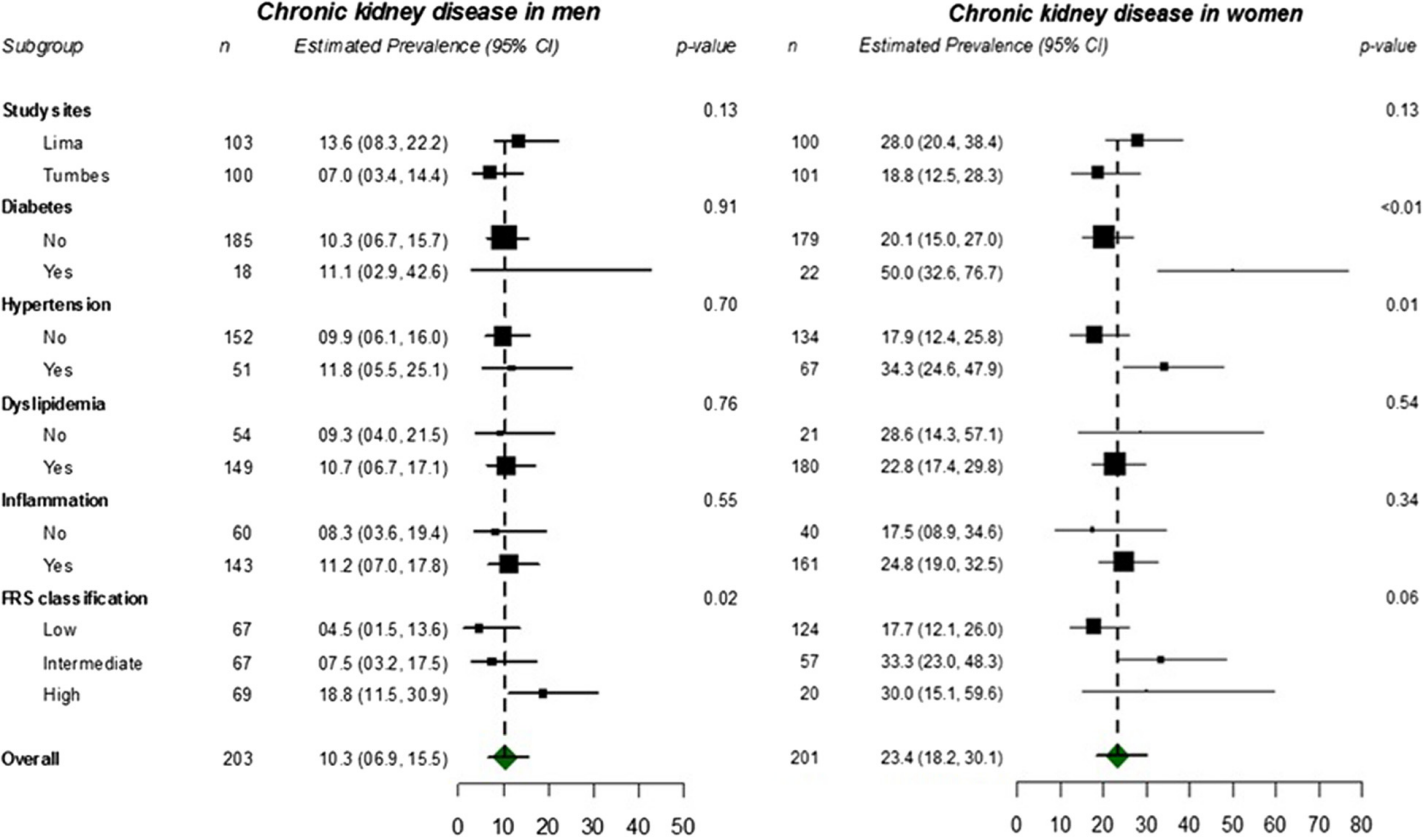
Differences between persons with and without chronic kidney disease stratified by sex

A higher prevalence of CKD was observed among females overall as well as within each age stratum (Table 3, Fig. 2). Among men, the prevalence of CKD was similar between most subgroups evaluated. The only exception was a tendency towards a gradient of doubling prevalence estimates in the Framingham risk score groups (Fig. 3, see also Additional file 1). In women, both diabetes and hypertension were associated with double the prevalence of CKD contrasted to those without these conditions (Fig. 3, see also Additional file 1).

**Table 3:** Prevalence of chronic kidney diseases stratified by age categories and sex

	Male		Female	
Age, years	Case/n	Estimated prevalence (95% CI)	Case/n	Estimated prevalence (95% CI)
35–44	3/51	5.8 (1.9–17.8)	5/51	9.8 (4.2–22.7)
45–54	5/54	9.2 (4.0–21.5)	12/51	23.5 (14.3–38.8)
55–64	6/51	11.8 (5.5–25.1)	9/48	18.8 (10.3–34.0)
≥65	7/47	14.9 (7.4–29.7)	21/51	41.2 (29.6–57.3)
Overall	21/203	10.3 (6.9–15.5)	47/201	23.4 (18.2–30.1)
*p*-value for trend	0.13		0.001

**Fig. 4:**
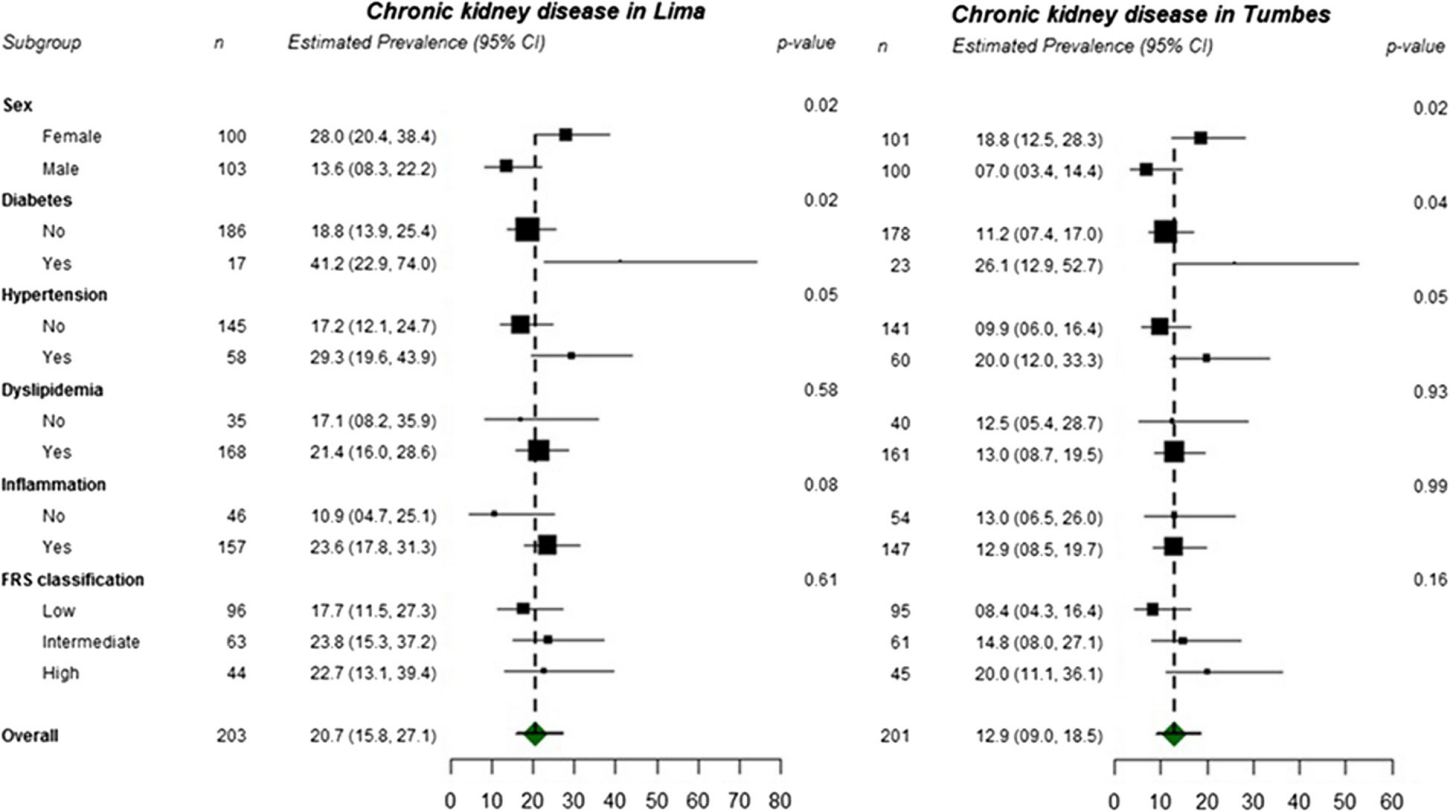
Differences between persons with and without chronic kidney disease stratified by geographical site

CKD prevalence was higher (p-value = 0.04) in Lima (21 %) than in Tumbes (13 %). Females, having diabetes, and hypertension were significantly associated with higher CKD prevalence in both sites (Fig. 4, see also Additional file 1).

## Discussion

In this representative cross-sectional sample of Peruvian adults from two different geographical sites, the prevalence of CKD was 16.8 %. These estimates are similar to those from high-income countries. Proteinuria, rather than reduced eGFR, was the defining factor for CKD in the vast majority (88 %) of individuals detected to have disease. In our sample, individuals with CKD were more likely to be female, older, of a higher socioeconomic stratum, diabetic and hypertensive. Compared to Tumbes, the study population in Lima had a higher prevalence of CKD.

Previous studies in Iran and the United States have found CKD prevalence estimates ranging between 13–18 %, and in Asian countries, such as China, Japan and Korea, 14–22 % [29–33]. Although measures of CKD prevalence in Latin America are rare [34], reports from El Salvador and Nicaragua have estimated that 12.7 % of these populations are afflicted with CKD [35, 36]. In addition, a recent survey examining CKD prevalence in Latin America reported the prevalence of proteinuria in Mexico and Chile to be 9.2 % and 14.2 %, respectively [37]. Notably, the definition of CKD used in these studies has varied including differences in thresholds defining proteinuria and eGFR, in equations used to calculate eGFR, and in the reliance on eGFR and proteinuria to define CKD. We defined CKD in accordance with other epidemiological studies by considering functional abnormalities, such as proteinuria, with and without lowered eGFR and utilizing prototypical cutoff values for both variables. We were not, however, able to incorporate the requirement of persistence of abnormalities for greater than three months [38].

Also noteworthy is the variability in the predominating factor defining CKD across diverse national settings- proteinuria dominance versus eGFR dominance. For instance, in Nicaragua, proteinuria was the predominant factor seen among CKD patients; an uncommon trend given that eGFR is usually more prominent [36]. In our study, CKD was proteinuria driven.

Older age and female gender were independent predictors of CKD among our participants, consistent with reports from the United States and Iran [29, 30] but not from Nicaragua and Sri Lanka where males had a higher prevalence of disease. CKD prevalence was higher in patients with vascular disease risk factors like diabetes, particularly in female patients and older patients. Indeed, in our female sample, but not in males, CKD was more prevalent among those who had diabetes and hypertension. It is noted however that males had a higher percentage of diabetes and hypertension, compared to females. The mechanisms underlying the sex difference in CKD epidemiology and CKD progression remain unclear and may involve a differential impact of traditional risk factors and environmental influences [39].

Poverty and social deprivation are documented risk factors for the development and progression rate of CKD in high-income countries and LMIC [40]. The sites fall below average socioeconomic indicators for Peru and Lima. For example, access to health insurance in our Lima and Tumbes sites was 37 % and 50 %, respectively [41]. Of note, the type of insurance reported is likely government subsidized insurance schemes, which cover basic services such as immunizations, maternal health, and large infectious diseases programs such as tuberculosis and HIV [9]. This could infer that access to general medical care where CKD can be detected and managed is low. Within this socioeconomic setting, and compared to the group with lowest wealth index, we identified a higher prevalence of CKD among participants within the highest wealth index tertile (all wealth strata were below the poverty line). Following significant expansion of Seguro Integral de Salud in 2009, it was noted that among the Peruvian poor, the poorest of the poor were insured to a greater extent than the wealthier of the poor [Ref is same as the new ref inserted in Intro]. Perhaps this subgroup accessed care that lessened the extent of CKD risk factors or, perhaps survival bias contributed to the trend. This difference, however, was not observed by education level.

CKD prevalence was higher in Lima than in Tumbes by nearly two-fold. This difference could be partly related to the higher prevalence of diabetes and hypertension, among patients with CKD, in Lima compared to Tumbes. It has been suggested that certain environmental factors promote CKD, including pesticide exposure, and others slow progression of CKD, including high altitude [35, 42, 43]. We were not able to directly examine either of these attributes in this study. More specifically, high altitude has been associated with higher levels of eGFR [43]. Though Lima and Tumbes are both approximately at sea level, nearly 50–60 % of the population at the Lima site are within-country migrants originating largely from high altitude Andean locations [41]. This might partially explain the smaller number of individuals in Lima with low eGFR and their higher average eGFR, compared to Tumbes. However, the reasons for the two-fold greater number of participants with proteinuria in Lima compared to Tumbes are unclear.

### Study strengths and limitations

The strengths of this study include high-quality data collection methods and surveillance of disease outcomes, all nested in a well-designed population-based study, a high response rate within the ancillary CKD component of the larger study, and standardized laboratory methods for measuring renal biomarkers. However, the study also had several limitations. First, while the cross-sectional design of this study provided the opportunity to estimate CKD prevalence in two Peruvian sites, causal relationships between CKD and measured risk factors cannot be determined. Further, not all potential risk factors were measured such as environmental exposures and health care accessibility. Second, misclassification of CKD status could not be excluded as we relied on a single measurement of kidney biomarkers rather than multiple measurements over time. Third, the sample size was moderate and may not have been sufficient for detection of small-to-moderate effects of hypothesized risk factors. Finally, some information biases related to the nature of the study cannot be discounted, such as temporal ambiguity and lead time biases [44].

## Conclusions

In summary, this study has identified a high prevalence, most likely undiagnosed, of CKD in Lima and Tumbes. This trend may or may not be representative of CKD throughout Peru. CKD’s silent progression, its association with cardiovascular disease, and high treatment costs make this disease one of great public health concern. As such, every effort should be made to expand upon this study through larger nationwide surveillance efforts. This will inform policies aimed at preventing CKD from escalating in Peru, especially given that renal replacement therapy is out of financial reach for the majority of Peruvians under their current health care system. These actions would pave the way for interventions aimed at reducing CKD prevalence and effectively managing existing cases of disease.

## Additional file

Additional file 1:
**Estimated prevalence of CKD and 95 % confidence interval by participants’ comorbidities stratified by sex and by area.**

## Abbreviations

CKD: Chronic kidney disease
CRONICAS: CRONICAS Center of Excellence in Chronic Disease
LMIC: Low- and middle-income countries
eGFR: Estimated glomerular filtration rate
SBP: Systolic blood pressure
DBP: Diastolic blood pressure
EDTA: Ethylenediaminetetraacetic acid
KDIGO: Kidney Disease Improving Global Outcomes
FRS: Framingham risk score
NCEP: National Cholesterol Education Program
ATP: Adult Treatment Panel
HOMA-IR: Homeostatic model assessment - Insulin Resistance
HDL: High-density lipoprotein cholesterol
CRP: C-reactive protein
BMI: Body mass index
IQR: Interquartile range
