# Supplementary material for: Burden of chronic kidney disease in resource-limited settings from Peru: a population-based study
Source: BMC Nephrol. 2015 Jul 24;16:114. doi: 10.1186/s12882-015-0104-7 (PMC4512019; doi:10.1186/s12882-015-0104-7)
Supplement: Additional file 1: — Estimated prevalence of CKD and 95 % confidence interval by participants’ comorbidities stratified by sex and by area. [file 12882_2015_104_MOESM1_ESM.docx]

## Additional Files

## Appendix 1. Estimated prevalence of CKD and 95% confidence interval by participants’ comorbidities stratified by sex

| **Subgroup** | **n** | **Male** | ***P* value** | **n** | **Female** | ***P* value** |
| --- | --- | --- | --- | --- | --- | --- |
| **Study site** |  |  |  |  |  |  |
| Lima | 103 | 13.6 (8.3-22.2) | 0.13 | 100 | 28.0 (20.4-38.4) | 0.13 |
| Tumbes | 100 | 7.0 (3.4-14.4) |  | 101 | 18.8 (12.5-28.3) |  |
| **Diabetes** |  |  |  |  |  |  |
| No | 185 | 10.3 (6.7-15.7) | 0.91 | 179 | 20.1 (15.0-27.0) | <0.01 |
| Yes | 18 | 11.1 (2.9-42.6) |  | 22 | 50.0 (32.6-76.7) |  |
| **Hypertension** |  |  |  |  |  |  |
| No | 152 | 9.9 (6.1-16.0) | 0.70 | 134 | 17.9 (12.4-25.8) | 0.01 |
| Yes | 51 | 11.8 (5.5-25.1) |  | 67 | 34.3 (24.6-47.9) |  |
| **Dyslipidemia** |  |  |  |  |  |  |
| No | 54 | 9.3 (4.0-21.5) | 0.76 | 21 | 28.6 (14.3-57.1) | 0.54 |
| Yes | 149 | 10.7 (6.7-17.1) |  | 180 | 22.8 (17.4-29.8) |  |
| **Inflammation (hsCRP >1 mg/dL)** |  |  |  |  |  |  |
| No | 60 | 8.3 (3.6-19.4) | 0.55 | 40 | 17.5 (8.9-34.6) | 0.34 |
| Yes | 143 | 11.2 (7.0-17.8) |  | 161 | 24.8 (19.0-32.5) |  |
| **Framingham risk score classification** |  |  |  |  |  |  |
| Low | 67 | 4.5 (1.5-13.6) | 0.02 | 124 | 17.7 (12.1-26.0) | 0.06 |
| Intermediate | 67 | 7.5 (3.2-17.5) |  | 57 | 33.3 (23.0-48.3) |  |
| High | 69 | 18.8 (11.5-30.9) |  | 20 | 30.0 (15.1-59.6) |  |
| **Overall** | 203 | 10.3 (6.9-15.5) |  | 201 | 23.4 (18.2-30.1) |  |

## Appendix 2. Estimated prevalence of CKD and 95% confidence interval by participants’ comorbidities stratified by area

| **Subgroup** | **n** | **Lima** | ***P* value** | **n** | **Tumbes** | ***P* value** |
| --- | --- | --- | --- | --- | --- | --- |
| **Sex** |  |  |  |  |  |  |
| Female | 100 | 28.0 (20.4-38.4) | 0.02 | 101 | 18.8 (12.5-28.3) | 0.02 |
| Male | 103 | 13.6 (8.3-22.2) |  | 100 | 7.0 (3.4-14.4) |  |
| **Diabetes** |  |  |  |  |  |  |
| No | 186 | 18.8 (13.9-25.4) | 0.02 | 178 | 11.2 (7.4-17.0) | 0.04 |
| Yes | 17 | 41.2 (22.9-74.0) |  | 23 | 26.1 (12.9-52.7) |  |
| **Hypertension** |  |  |  |  |  |  |
| No | 145 | 17.2 (12.1-24.7) | 0.05 | 141 | 9.9 (6.0-16.4) | 0.05 |
| Yes | 58 | 29.3 (19.6-43.9) |  | 60 | 20.0 (12.0-33.3) |  |
| **Dyslipidemia** |  |  |  |  |  |  |
| No | 35 | 17.1 (8.2-35.9) | 0.58 | 40 | 12.5 (5.4-28.7) | 0.93 |
| Yes | 168 | 21.4 (16.0-28.6) |  | 161 | 13.0 (8.7-19.5) |  |
| **Inflammation (hsCRP >1 mg/dL)** |  |  |  |  |  |  |
| No | 46 | 10.9 (4.7-25.1) | 0.08 | 54 | 13.0 (6.5-26.0) | 0.99 |
| Yes | 157 | 23.6 (17.8-31.3) |  | 147 | 12.9 (8.5-19.7) |  |
| **Framingham risk score classification** |  |  |  |  |  |  |
| Low | 96 | 17.7 (11.5-27.3) | 0.61 | 95 | 8.4 (4.3-16.4) | 0.16 |
| Intermediate | 63 | 23.8 (15.3-37.2) |  | 61 | 14.8 (8.0-27.1) |  |
| High | 44 | 22.7 (13.1-39.4) |  | 45 | 20.0 (11.1-36.1) |  |
| **Overall** | 203 | 20.7 (15.8-27.1) |  | 201 | 12.9 (9.0-18.5) |  |
